# Non–AIDS‐Defining Cancer Incidence in People With HIV in Milan, Italy, 2000–2023: Temporal Trends and Comparison With the General Population

**DOI:** 10.1002/ijc.70689

**Published:** 2026-08-07

**Authors:** Giacomo Pozza, Maddalena Matone, Letizia Oreni, Giorgia Carrozzo, Beatrice Caloni, Maria Vittoria Cossu, Davide Moschese, Anna Lisa Ridolfo, Cristina Gervasoni, Cinzia Fasola, Nicla La Verde, Davide Dalu, Spinello Antinori, Andrea Gori, Andrea Giacomelli

**Affiliations:** ^1^ Department of Infectious Diseases Luigi Sacco Hospital, ASST Fatebenefratelli Sacco Milan Italy; ^2^ Department of Biomedical and Clinical Sciences Università degli Studi di Milano Milan Italy; ^3^ Department of Oncology ASST Fatebenefratelli‐Sacco, Luigi Sacco University Hospital Milan Italy; ^4^ Multidisciplinary Action for Global Health (MAGH), University of Milan Milan Italy

**Keywords:** epidemiology, HIV, incidence, non–AIDS‐defining cancers, standardized incidence ratio

## Abstract

People with HIV (PWH) are living longer, but contemporary data on non–AIDS‐defining cancers (NADCs) in Italy remain limited. We assessed incident cancers in a retrospective cohort of PWH in Milan, Italy, between January 2000 and December 2023. Follow‐up was grouped into 2000–2007, 2008–2015, and 2016–2023. Incidence rates (IRs) were calculated per 100,000 person‐years (PYs). Multivariable Poisson regression estimated adjusted (age, mode of HIV acquisition, and prior AIDS diagnosis) IR ratios (aIRRs) for first incident NADCs. Standardized incidence ratios (SIRs) compared observed cancers with expected cases in the general population. Among 4642 PWH contributing 50,086 PYs, 321 first incident NADCs were diagnosed. NADC incidence increased from 518 (95% CI 422–636) in 2000–2007 to 812 (95% CI 683–967) in 2016–2023. Non–virus‐related cancer incidence increased from 293 to 582 per 100,000 PYs, whereas virus‐related cancer incidence remained stable, from 225 to 230 per 100,000 PYs. People who inject drugs (PWID) versus heterosexuals (aIRR 1.71, 95% CI 1.33–2.20), and prior AIDS diagnosis (aIRR 1.33, 95% CI 1.05–1.69) were associated with higher NADC incidence. Compared with the general population, lung cancer incidence was increased in females (SIR 3.21, 95% CI 1.94–5.32). Anal cancer incidence was markedly elevated in both males and females (SIR 59.16, 95% CI 42.06–83.22 and 18.55, 95% CI 8.33–41.30, respectively). The excess cancer burden in this cohort was concentrated in selected NADCs, particularly anal cancer and, in females, lung cancer.

## What's New?

As people with HIV live longer, the burden of non‐AIDS‐defining cancers (NADCs)—malignancies not traditionally associated with HIV infection—is increasing. Data on NADC incidence and risk patterns, however, remain limited. Here, among people with HIV followed over more than 20 years, the authors evaluated trends in NADC incidence and risk. NADC incidence increased noticeably over time, driven by non‐HIV‐related cancers, whereas virus‐related cancer rates were relatively unchanged. Anal cancer risk remained markedly elevated in both sexes, and excess lung cancer risk was observed only in females. The findings identify priorities for targeted cancer prevention and screening within routine HIV care.

## Graphical abstract

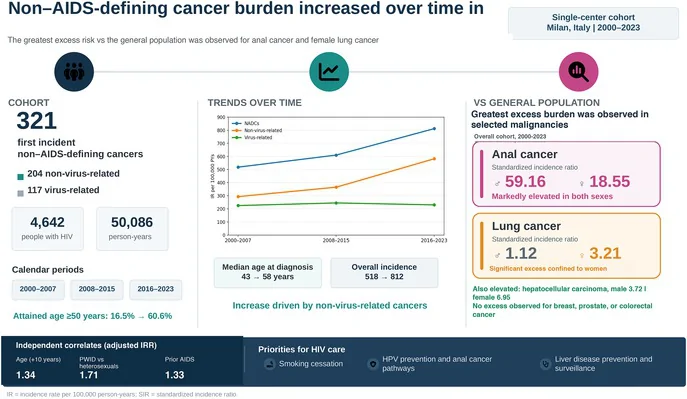

**Abbreviations**
ADC: AIDS‐defining cancer
AIDS: acquired immunodeficiency syndrome
aIRR: adjusted incidence rate ratio
ART: antiretroviral therapy
cART: combination antiretroviral therapy
CI: confidence interval
HBV: hepatitis B virus
HCV: hepatitis C virus
HE: heterosexual contact
HIV: human immunodeficiency virus
HPV: human papillomavirus
IARC: International Agency for Research on Cancer
ICD‐10: International Classification of Diseases, 10th Revision
ICD‐O‐3: International Classification of Diseases for Oncology, 3rd Edition
INSTI: integrase strand transfer inhibitor
IR: incidence rate
MSM: men who have sex with men
NADC: non–AIDS‐defining cancer
PWH: people with HIV
PWID: people who inject drugs
PY: person‐year
SIR: standardized incidence ratio

## 1 Introduction

The introduction of combination antiretroviral therapy (cART) since the mid‐1990s has markedly reduced AIDS‐related morbidity and mortality, leading to a substantial increase in life expectancy among people with HIV (PWH), now approaching that of the general population [1]. As a consequence, the aging of PWH has been accompanied by a growing burden of non‐communicable diseases, including cancer [2].

Over time, the spectrum of malignancies in PWH has shifted. While the incidence of AIDS‐defining cancers (ADCs), such as Kaposi sarcoma and non‐Hodgkin lymphoma, has declined, non‐AIDS‐defining cancers (NADCs) have become increasingly prominent [3]. This trend reflects a multifactorial pathogenesis involving persistent immune dysregulation and inflammation, incomplete immune restoration despite cART, and a higher prevalence of oncogenic viral co‐infections (e.g., HBV, HCV, HPV) and behavioral risk factors such as smoking and alcohol use [4, 5, 6].

In Italy, cohort studies have shown a post‐cART decline in ADCs, alongside persistently elevated rates of several NADCs, including liver, lung, cervical, and anal cancers [7, 8]. More recent data from ICONA cohort confirm that, despite widespread cART use, PWH continue to experience a higher cancer incidence compared with the general population, underscoring the need for targeted prevention, and screening strategies [9]. Despite substantial declines in AIDS‐related mortality in the ART era, non‐AIDS‐related conditions, particularly cancers, have become leading causes of death among PWH, reflecting the impact of aging and long‐term comorbidities [10]. However, contemporary data on temporal trends and clinical determinants of NADCs in Italian real‐world cohorts remain limited, particularly in the most recent cART era.

In this context, we aimed to evaluate the incidence of cancers in PWH over a 23‐year period at a single Italian center.

## 2 Materials and Methods

### 2.1 Study Design

We conducted a retrospective observational study including a cohort of PWH receiving care at the Infectious Diseases Unit of Luigi Sacco Hospital in Milan, Italy, between January 2000 and December 2023. The study population included PWH with access to regular care and monitoring, defined as having at least one follow‐up visit and a minimum observation period of ≥ 6 months. PWH with a diagnosis of NADC prior to cohort entry were excluded from the analysis.

### 2.2 Data Collection and Variables

Data were retrospectively collected from electronic medical records, including clinical registries containing demographic information, vital status, medical history, comorbidities, treatments, diagnostic tests, imaging results, and histopathological reports. Virological and immunological characteristics were also collected, including mode of HIV acquisition and AIDS‐defining events.

All solid and hematological malignancies were considered. Cancer sites and morphologies were coded according to the International Classification of Diseases for Oncology, 3rd Edition (World Health Organization, 2000), and the 10th Revision of the International Classification of Diseases and Related Health Problems (World Health Organization, 2019) [11].

Cancers were classified as ADCs or NADCs (Table S1) [12]. Furthermore, NADCs were subdivided into virus‐related and non‐virus‐related cancers (Table S2) [12].

### 2.3 Statistical Analysis

The event of interest was defined as the diagnosis of a first incident NADC during follow‐up; only the first event was considered.

Descriptive statistical analyses were performed to summarize the demographic characteristics of the cohort. Cancer incidence was calculated as the number of new cases per 100,000 person‐years (PYs). The observation period was divided into three 8‐year calendar periods: 2000–2007 (pre‐INSTI era), 2008–2015 (INSTI era before universal cART), and 2016–2023 (post–universal cART implementation) [13].

Incidence rates were estimated for overall NADCs and stratified by virus‐related and non‐virus‐related cancers.

Multivariable Poisson regression models were used to evaluate associations, expressed as adjusted incidence rate ratios (aIRRs), between calendar period, age, mode of HIV acquisition [heterosexual contact (HE), men who have sex with men (MSM), people who inject drugs (PWID), other/unknown], prior AIDS diagnosis, and cancer risk (overall NADCs, non‐virus‐related cancers, virus‐related cancers, lung cancer, hepatocellular carcinoma, and anal cancer).

Standardized incidence ratios (SIRs) were calculated for anal cancer, lung cancer, hepatocellular carcinoma, breast cancer, Hodgkin lymphoma, melanoma, prostate cancer, and colorectal cancer by dividing the observed number of cancer cases in the cohort by the expected number based on incidence rates in the general Italian population residing in the Milan metropolitan area. We chose local rather than national Italian incidence rates because the cohort was recruited from a Milan‐based clinical center and most participants resided in the Metropolitan City of Milan; this approach was intended to reduce potential bias due to geographic heterogeneity in background cancer incidence.

Specifically, 3516/4642 participants (76% of the overall cohort) resided in the Metropolitan City of Milan, while most of the remaining 24% resided in other provinces of Lombardy or comparable Northern Italian areas. To evaluate the robustness of this standardization strategy and the potential impact of including participants residing outside the Metropolitan City of Milan, we performed a sensitivity analysis restricted to Milan metropolitan residents only. The expected number of cases was calculated by multiplying PYs at risk by sex‐, age‐, and calendar period–specific cancer incidence rates derived from Italian cancer registries and published by the International Agency for Research on Cancer (IARC) in Cancer Incidence in Five Continents, volumes IX (1998–2002), X (2003–2007), XI (2008–2012), and XII (2013 onward) [14].

Ninety‐five percent confidence intervals (CIs) for SIRs were calculated assuming a Poisson distribution. All statistical analyses were performed using SAS software, version 9.4. Two‐sided *p* values < 0.05 were considered statistically significant.

## 3 Results

### 3.1 Study Population

A total of 4642 PWH were included, contributing 50,086 PYs (Table S3). Males accounted for 74.6% of the cohort. The proportion of individuals aged ≥ 50 years increased from 16.5% in 2000–2007 to 60.6% in 2016–2023, with those aged 60–69 rising from 4.8% to 19.6% and ≥ 70 years from 1.4% to 6.6%. Over time, HE and MSM increased (35.0%–42.1% and 24.9%–39.1%, respectively), while PWID declined (32.7%–14.8%).

### 3.2 Cancer Incidence

Overall, 534 first malignancies were observed, including 321 (60.1%) NADCs. NADC incidence increased from 518 (95% CI 422–636) to 812 (95% CI 683–967) per 100,000 PYs across study periods, with a parallel increase in median age at diagnosis (43–58 years) (Table 1).

**TABLE 1:** Incidence rate of NADCs, non‐virus‐related cancers, virus‐related cancers and specific type of cancer.

	Overall	2000–2007	2008–2015	2016–2023
NADCs, *n*	321	92	102	127
IR [95% CI] per 100,000 PYs	641 [574–715]	518 [422–636]	610 [503–741]	812 [683–967]
Age at the time of NADC development, median [IQR]	53 [44–59]	43 [39–49]	51 [46–58]	58 [53–62]
Non‐virus‐related cancers, *n*	204	52	61	91
IR [95% CI] per 100,000 PYs	407 [355–467]	293 [223–385]	365 [284–469]	582 [474–715]
Age at the time of cancer development, median [IQR]	55 [45–61]	42 [37–49]	55 [46–61]	59 [53–64]
Virus‐related cancers, *n*	117	40	41	36
IR [95% CI] per 100,000 PYs	234 [195–280]	225 [165–307]	245 [181–333]	230 [166–319]
Age at time of cancer development, median [IQR]	49 [44–55]	44 [40–52]	48 [46–52]	55 [51–59]
Lung cancer, *n*	51	13	13	25
IR [95% CI] per 100,000 person‐years	102 [77–134]	73 [42–126]	78 [45–134]	160 [108–237]
Age at time of cancer development, median [IQR]	55 [49–59]	46 [43–49]	56 [52–60]	57 [53–62]
Hepatocarcinoma, *n*	40	12	20	8
IR [95% CI] per 100,000 person‐years	80 [59–109]	68 [38–119]	120 [77–185]	51 [26–102]
Age at time of cancer development, median [IQR]	52 [46–55]	45 [41–55]	49 [48–53]	58 [54–62]
Anal cancer, *n*	39	11	10	18
IR [95% CI] per 100,000 person‐years	78 [57–107]	62 [34–112]	60 [32–111]	115 [73–183]
Age at time of cancer development, median [IQR]	50 [43–56]	43 [41–47]	48 [44–49]	56 [52–59]

Abbreviations: IQR, Inter Quartile Range; IR, incidence rate; *n*, number; PYs, person years.

NADCs rose in absolute numbers from 92 to 127 cases. Non–virus‐related cancers accounted for the majority (*n* = 204) and increased from 293 to 582 per 100,000 PYs, whereas virus‐related cancers (*n* = 117) remained stable (225–230 per 100,000 PYs).

Among site‐specific cancers, lung cancer incidence increased from 73 to 160 per 100,000 PYs (*n* = 13 to *n* = 25), and anal cancer from 62 to 115 per 100,000 PYs (*n* = 11 to *n* = 18). Hepatocellular carcinoma peaked in 2008–2015 (120 per 100,000 PYs; *n* = 20) and declined thereafter (51 per 100,000 PYs; *n* = 8).

### 3.3 Factors Associated With Cancer

Increasing age (per 10 years; aIRR 1.34, 95% CI 1.22–1.47), PWID (vs. HE; aIRR 1.71, 95% CI 1.33–2.20), and prior AIDS (aIRR 1.33, 95% CI 1.05–1.69) were associated with higher NADC incidence (Tables S4–S9).

Age was the main determinant of non–virus‐related cancers (aIRR 1.45, 95% CI 1.30–1.63), whereas PWID and prior AIDS were strongly associated with virus‐related cancers (aIRR 2.79 and 1.76, respectively). PWID showed a markedly increased risk of hepatocellular carcinoma (aIRR 11.16, 95% CI 4.28–29.08), while MSM and prior AIDS were associated with anal cancer (aIRR 2.20 and 4.07, respectively).

### 3.4 Standardized Incidence Ratios

Compared with the general population, lung cancer incidence was not increased in males (SIR 1.12, 95% CI 0.81–1.55) but was higher in females (SIR 3.21, 95% CI 1.94–5.32) (Figure 1A). Anal cancer incidence was markedly elevated in both males and females (SIR 59.16, 95% CI 42.06–83.22 and 18.55, 95% CI 8.33–41.30, respectively) (Figure 1B).

**FIGURE 1:**
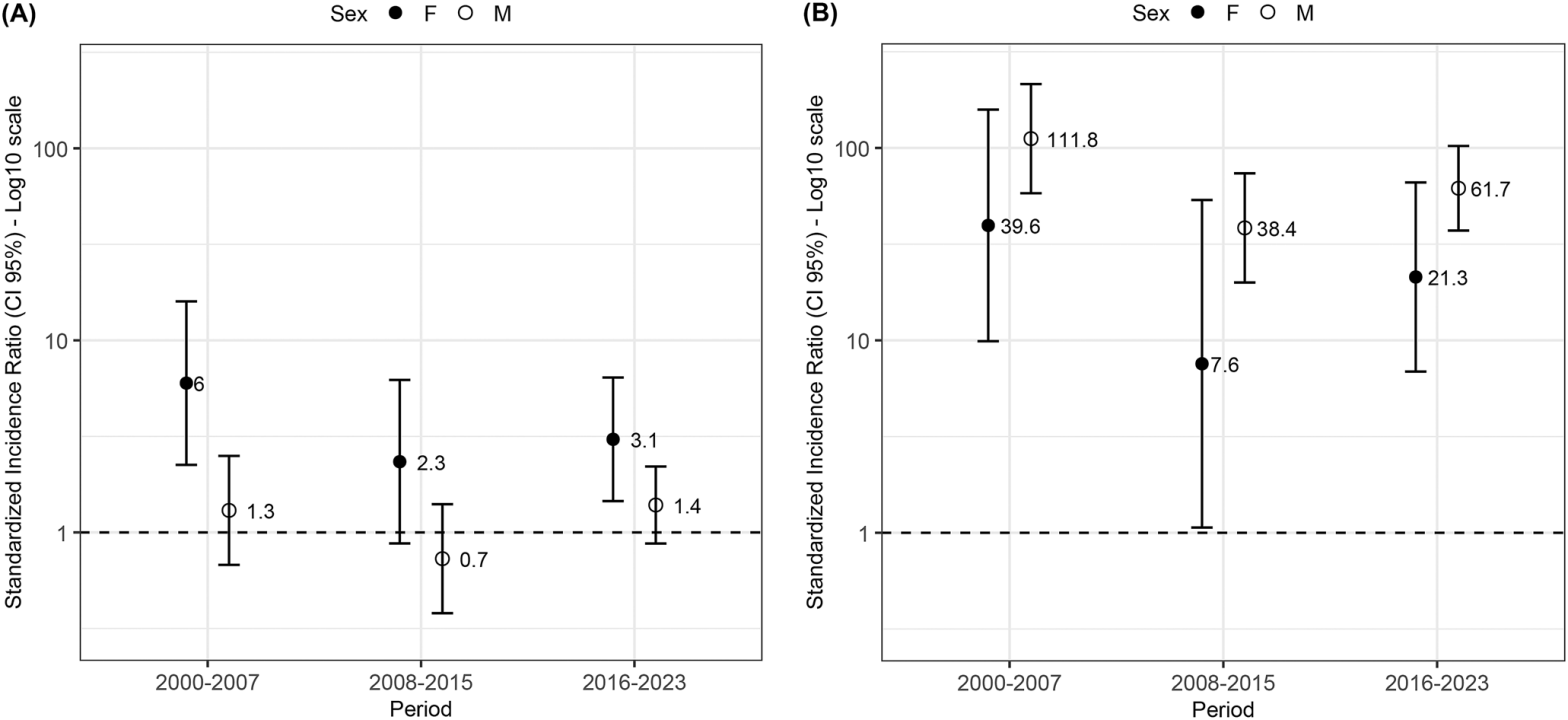
Standardized incidence ratios (SIRs) for lung cancer (A) and anal cancer (B) in people with HIV compared with the general population, stratified by sex and calendar period. Error bars indicate 95% confidence intervals.

Hepatocellular carcinoma incidence was increased in both sexes (males: SIR 3.72, 95% CI 2.67–5.18; females: SIR 6.95, 95% CI 2.89–16.69), as was Hodgkin lymphoma in males (SIR 10.05, 95% CI 6.25–16.16), while it could not be estimated in females due to the small number of cases.

Melanoma incidence did not differ significantly from that of the general population among either males or females (males: SIR 1.20, 95% CI 0.64–2.22; females: SIR 1.07, 95% CI 0.34–3.31). Similarly, breast cancer incidence was not significantly different from that of the general population (SIR 0.87, 95% CI 0.58–1.31). In contrast, prostate cancer (SIR 0.30, 95% CI 0.18–0.51) and colorectal cancer among males (SIR 0.53, 95% CI 0.31–0.89) showed a lower incidence compared with the general population, whereas no significant difference was observed for colorectal cancer among females (SIR 0.71, 95% CI 0.27–1.90).

Regarding sensitivity analysis restricting the cohort of 3516 residents of Metropolitan City of Milan (76% of the overall cohort) the SIRs were consistent with the results of the primary analysis (Table S10).

## 4 Discussion

In this study, we observed an increasing incidence of NADCs over time, particularly for non–virus‐related cancers and for lung and anal cancer. These findings are consistent with previous studies showing a growing burden of malignancies in aging PWH [15].

Persistent immune dysregulation, oncogenic viral coinfections, and behavioral risk factors contribute to cancer risk in PWH, even in the ART era [16]. In our cohort, prior AIDS diagnosis remained associated with NADC incidence, supporting the role of immunosuppression in cancer development, particularly for virus‐related malignancies [16]. Unlike previous reports suggesting a decline in virus‐related cancers [17, 18], we observed broadly stable rates over time, although hepatocellular carcinoma declined in the most recent period, likely reflecting the impact of direct‐acting antivirals for HCV and widespread HBV vaccination and treatment [18].

Compared with the ICONA cohort [9], the largest Italian study evaluating cancer risk in PWH, our study offers complementary information, reflecting a real‐world population with long‐term follow‐up and a high proportion of older and treatment‐experienced individuals. In the most recent calendar period, approximately 61% of participants were aged ≥ 50 years, and the cohort contributed 50,086 PYs, with 534 first malignancies observed (vs. 763 events over 133,851 PYs in ICONA). These differences in population structure, partly explained by ICONA's enrollment of ART‐naive PWH, should be considered when comparing incidence rates across studies. In particular, the significantly higher hepatocellular carcinoma SIR observed in our cohort compared with ICONA, in both sexes, likely reflects this difference in case mix, including a higher proportion of PWID in our population. Several elements distinguish our findings from previous ICONA data. First, we show that the rise of NADC over time is driven mainly by non–virus‐related cancers, reflecting an ongoing epidemiological transition not captured by ICONA's cross‐sectional comparison with the general population. Second, multivariable Poisson regression, an analysis not performed in ICONA, identified independent determinants of cancer risk, including older age, PWID status, and prior AIDS diagnosis, as well as site‐specific associations, such as PWID with hepatocellular carcinoma [18] and MSM/prior AIDS with anal cancer [19]. Third, our sex‐stratified SIR estimates show that excess lung cancer risk was confined to females (SIR 3.21), while anal cancer was elevated in both sexes, more markedly in males (SIR 59.16 and 18.55, respectively). Of note, the anal cancer SIR in ICONA could not be computed for females due to the paucity of events. Finally, we used local (Milan metropolitan area) rather than national reference rates for SIR calculation, reducing geographic confounding; a sensitivity analysis restricted to Milan residents yielded results consistent with the primary analysis.

Anal cancer incidence was markedly elevated compared with the general population, particularly among men, but also among women. While previous studies have mainly focused on men [19], our findings highlight a substantial burden also in women. This likely reflects persistent HPV infection and incomplete immune recovery despite ART, compounded by suboptimal implementation of screening programs [18, 20, 21], as well as additional behavioral factors such as smoking rather than HIV infection alone [17].

Lung cancer incidence increased over time, particularly in older individuals, consistent with data from other cohorts [22]. Smoking remains the main driver, although HIV‐related factors such as chronic inflammation and immune dysregulation may contribute [23]. Notably, we observed a higher SIR for lung cancer in women than in men, in line with previous evidence suggesting increased susceptibility among women with HIV [24].

Conversely, prostate, breast, and colorectal cancers showed lower incidence compared with the general population, as previously reported, possibly reflecting a combination of biological mechanisms and differences in screening uptake [25].

This study has several limitations. First, its retrospective single‐center design and the limited number of events for some cancer sites may reduce statistical power and generalizability. Second, our analyses were not adjusted for time‐updated HIV‐related variables, including CD4 cell count, HIV‐RNA, or specific antiretroviral regimens. Because calendar period was the main exposure of interest, changes in antiretroviral treatment, immune‐virological control, and clinical management over time were considered part of the broader temporal effect rather than separately modeled as independent predictors. The absence of time‐updated CD4, HIV‐RNA, and ART exposure data limits causal interpretation of our findings, as CD4 cell count is a well‐established predictor of cancer risk: data from the D:A:D/RESPOND collaboration showed that, even among PWH with sustained virological suppression for more than 2 years, those with poorer immune recovery (CD4 < 350 cells/μL) had a significantly higher cancer incidence than those achieving CD4 ≥ 750 cells/μL [12]. Third, individual‐level data on smoking and alcohol consumption were not systematically collected over the entire 23‐year study period. This is particularly relevant for lung cancer and hepatocellular carcinoma, for which behavioral risk factors may substantially contribute to carcinogenic effects. Therefore, residual confounding by unmeasured HIV‐related and lifestyle factors cannot be excluded. Because behavioral factors are not routinely available in population‐based cancer registries, the SIR analyses could not account for differences in smoking or alcohol exposure between PWH and the reference population. Fourth, SIRs were standardized using cancer incidence rates from the Metropolitan City of Milan, whereas the cohort included all eligible PWH followed at Sacco Hospital and was not restricted to Milan metropolitan residents. Although this choice may have introduced some geographic misclassification in expected cancer rates, most participants resided in the Metropolitan City of Milan, and sensitivity analyses restricted to Milan metropolitan residents yielded estimates consistent with the primary analysis. Finally, cancer ascertainment was based on clinical follow‐up and cancer diagnosis was based on histopathological reports. Individuals lost to follow‐up were censored at the date of their last clinical visit, and PYs were accrued until that time point; as no formal linkage with a population‐based cancer registry was available, some under‐ascertainment of cancers diagnosed after loss to follow‐up cannot be excluded, and completeness could not be formally quantified.

## 5 Conclusions

Between 2000 and 2023, cancer incidence in PWH increased, driven by aging and a rise in NADCs, particularly lung and anal cancers. Prior AIDS diagnosis remained associated with cancer risk, highlighting the role of immunosuppression. Notably, lung cancer incidence was disproportionately high among women. These findings support the need for integrated cancer prevention strategies in PWH, including early ART initiation, targeted screening, vaccination, and behavioral interventions such as smoking cessation.

## Author Contributions

**Giacomo Pozza:** conceptualization, investigation, writing – original draft. **Maddalena Matone:** investigation, writing – review and editing. **Letizia Oreni:** methodology, data curation, formal analysis, writing – review and editing. **Giorgia Carrozzo:** investigation, writing – review and editing. **Beatrice Caloni:** investigation, writing – review and editing. **Maria Vittoria Cossu:** methodology, investigation, writing – review and editing. **Davide Moschese:** investigation, writing – review and editing. **Anna Lisa Ridolfo:** methodology, investigation, writing – review and editing, supervision. **Cristina Gervasoni:** methodology, investigation, writing – review and editing, supervision. **Cinzia Fasola:** investigation, writing – review and editing. **Nicla La Verde:** investigation, writing – review and editing. **Davide Dalu:** investigation, writing – review and editing. **Spinello Antinori:** supervision, writing – review and editing. **Andrea Gori:** supervision, writing – review and editing. **Andrea Giacomelli:** conceptualization, methodology, investigation, supervision, writing – review and editing.

## Ethics Statement

The study was approved by our local Ethics Committee (IRB approval No. CET 394‐2025; 01/16/2026) and informed consent was obtained for each patient.

## Conflicts of Interest

The authors declare no conflicts of interest.

## Supporting information


**Table S1:** List of AIDS and non‐AIDS related cancers observed during the study period.
**Table S2:** List of virus and non‐virus related NADCs.
**Table S3:** Characteristics of study population.
**Table S4:** Multivariable Poisson regression models of risk of development NADCs.
**Table S5:** Multivariable Poisson regression models of risk of development non‐virus‐related cancers.
**Table S6:** Multivariable Poisson regression models of risk of development virus‐related cancers.
**Table S7:** Multivariable Poisson regression models of risk of development lung cancers.
**Table S8:** Multivariable Poisson regression models of risk of development hepatocarcinoma.
**Table S9:** Multivariable Poisson regression models of risk of development anal cancer.
**Table S10:** Standardized incidence ratios (SIRs) for selected cancers in the overall cohort and in the sensitivity analysis restricted to participants residing in the Metropolitan City of Milan. Estimates are shown with 95% confidence intervals.

## Data Availability

The data that support the findings of this study are available from the corresponding author upon reasonable request.
