# Supplementary material for: Non–AIDS‐Defining Cancer Incidence in People With HIV in Milan, Italy, 2000–2023: Temporal Trends and Comparison With the General Population
Source: Int J Cancer. 2026 Aug 7;159(12):2870–6. doi: 10.1002/ijc.70689 (PMC13688222; doi:10.1002/ijc.70689)
Supplement: Supplementary file 1 [file IJC-159-2870-s001.pdf]

Non–AIDS-defining cancer incidence in people with HIV in Milan, Italy, 2000–2023: temporal trends and comparison with the general population

Giacomo Pozza, Maddalena Matone, Letizia Oreni, Giorgia Carrozzo, Beatrice Caloni, Maria Vittoria Cossu, Davide Moschese, Anna Lisa Ridolfo, Cristina Gervasoni, Cinzia Fasola, Nicla La Verde, Davide Dalu, Spinello Antinori, Andrea Gori, and Andrea Giacomelli.

Table of Contents

**Supplementary Tables**	Page
Supplementary Table 1. List of AIDS and non AIDS related cancers observed during the study period.	2
Supplementary Table 2. List of Virus and Non-Virus related NADCs.	3
Supplementary Table 3. Characteristics of study population.	4
Supplementary Table 4. Multivariable Poisson regression models of risk of development NADCs.	4
Supplementary Table 5. Multivariable Poisson regression models of risk of development non-virus-related cancers.	5
Supplementary Table 6. Multivariable Poisson regression models of risk of development virus-related cancers.	5
Supplementary Table 7. Multivariable Poisson regression models of risk of development lung cancers.	6
Supplementary Table 8. Multivariable Poisson regression models of risk of development hepatocarcinoma.	6
Supplementary Table 9. Multivariable Poisson regression models of risk of development anal cancer.	7
Supplementary Table 10. Standardized incidence ratios (SIRs) for selected cancers in the overall cohort and in the sensitivity analysis restricted to participants residing in the Metropolitan City of Milan. Estimates are shown with 95% confidence intervals.	7

**AIDS-related cancers (n=213)**	**Non-AIDS-related cancers (n=321)**
Kaposi sarcoma (n=99)<br>Non-Hodgkin lymphoma (n=98)<br>Invasive cervical cancer (n=13)<br>Castleman disease/serosal lymphoma (n=3)	Lung cancer (n=51)<br>Hepatocellular carcinoma (n=40)<br>Anal cancer (n=39)<br>Breast cancer (n=25)<br>Colon/rectal cancer (n=18)<br>Hodgkin lymphoma (n=18)<br>Prostate cancer (n=15)<br>Melanoma (n=13)<br>Bladder cancer (n=13)<br>Oropharyngeal cancer (n=12)<br>Pancreatic cancer (n=10)<br>Head and neck cancer (n=8)<br>Cholangiocarcinoma (n=6)<br>Kidney cancer (n=6)<br>Endometrial cancer (n=5)<br>Thyroid cancer (n=5)<br>Vulvar cancer (n=4)<br>Ovarian cancer (n=4)<br>Penile cancer (n=4)<br>Gastric cancer (n=4)<br>Brain cancer (n=4)<br>Seminoma (n=4)<br>Esophageal cancer (n=3)<br>Laryngeal cancer (n=3)<br>Adrenal gland malignancy (n=2)<br>Leukemia (n=2)<br>Primary bone cancer (n=1)<br>Neuroendocrine tumor (n=1)<br>Soft tissue sarcoma (n=1)

Supplementary Table 1. List of AIDS and non AIDS related cancers observed during the study period.

**Virus-related cancers (n=117)**	**Non-virus-related cancers (n=204)**
Anal cancer (n=39)<br>Hepatocellular carcinoma (n=40)<br>Hodgkin lymphoma (n=18)<br>Oropharyngeal cancer (n=12)<br>Vulvar cancer (n=4)<br>Penile cancer (n=4)	Lung cancer (n=51)<br>Breast cancer (n=25)<br>Colon/rectal cancer (n=18)<br>Prostate cancer (n=15)<br>Melanoma (n=13)<br>Bladder cancer (n=13)<br>Pancreatic cancer (n=10)<br>Head and neck cancer (n=8)<br>Cholangiocarcinoma (n=6)<br>Kidney cancer (n=6)<br>Endometrial cancer (n=5)<br>Thyroid cancer (n=5)<br>Ovarian cancer (n=4)<br>Gastric cancer (n=4)<br>Brain cancer (n=4)<br>Seminoma (n=4)<br>Esophageal cancer (n=3)<br>Laryngeal cancer (n=3)<br>Adrenal gland malignancy (n=2)<br>Leukemia (n=2)<br>Primary bone cancer (n=1)<br>Neuroendocrine tumor (n=1)<br>Soft tissue sarcoma (n=1)

Supplementary Table 2. List of Virus and Non-Virus related NADCs.

Characteristics	Overall<br>N=4,642	2000-2007<br>N=3,467	2008-2015<br>N=2,822	2016-2023<br>N=2,547
**Person-years**	50,086	17,742	16,712	15,632
**Assigned sex at birth, n (%)**				
Male	3,461 (74.6)	2,505 (72.3)	2,067 (73.2)	1,890 (74.2)
Female	1,181 (25.4)	962 (27.7)	755 (26.8)	657 (25.8)
**Mode of HIV acquisition, n (%)**				
HE	1,676 (36.1)	1,213 (35)	1,137 (40.3)	1,072 (42.1)
MSM	1,527 (32.9)	863 (24.9)	982 (34.8)	997 (39.1)
PWID	1159 (25)	1135 (32.7)	571 (20.2)	378 (14.8)
Other/unknown	280 (6)	256 (7.4)	132 (4.7)	100 (3.9)
**Attained age group, n (%)**				
<40	-	1390 (40.1)	665 (23.6)	467 (18.4)
40-49	-	1507 (43.5)	1001 (35.5)	536 (21)
50-59	-	358 (10.3)	861 (30.5)	879 (34.5)
60-69	-	165 (4.8)	195 (6.9)	498 (19.6)
70+	-	47 (1.4)	100 (3.5)	167 (6.6)
**Prior AIDS diagnosis, n (%)**	-	740 (21.3)	655 (23.2)	576 (22.6)

Supplementary Table 3. Characteristics of study population.
List of abbreviations: HE, heterosexual; MSM, man who have sex with man; PWID, people who inject drugs.

	aIRR (95% CI)	p
**Period**		
2008-2015 vs 2000-2007	1.06 (0.81-1.40)	0.666
2016-2023 vs 2000-2007	1.20 (0.90-1.59)	0.219
**Attained age (x 10 years more)**	1.34 (1.22-1.47)	<.0001
**HIV risk group**		
PWID vs HE	1.71 (1.33-2.20)	<.0001
MSM vs HE	1.04 (0.80-1.37)	0.757
Other/unknown vs HE	1.37 (0.84-2.23)	0.201
**Prior AIDS diagnosis, yes vs no**	1.33 (1.05-1.69)	0.016

Supplementary Table 4. Multivariable Poisson regression models of risk of development NADCs.

List of abbreviations: aRR, adjusted relative risk; CI, confidence interval; PWID, people who inject drugs; HE, heterosexual; MSM, man who have sex with man.

	aIRR (95% CI)	p
**Period**		
2008-2015 vs 2000-2007	1.01 (0.71-1.43)	0.968
2016-2023 vs 2000-2007	1.31 (0.92-1.85)	0.129
**Attained age (x 10 years more)**	1.45 (1.30-1.63)	<.0001
**HIV risk group**		
PWID vs HE	1.33 (0.97-1.82)	0.075
MSM vs HE	0.88 (0.63-1.23)	0.459
Other/unknown vs HE	1.30 (0.72-2.33)	0.386
**Prior AIDS diagnosis, yes vs no**	1.13 (0.84 -1.53)	0.404

Supplementary Table 5. Multivariable Poisson regression models of risk of development non-virus-related cancers.

List of abbreviations: aRR, adjusted relative risk; CI, confidence interval; PWID, people who inject drugs; HE, heterosexual; MSM, man who have sex with man.

	aIRR (95% CI)	p
**Period**		
2008-2015 vs 2000-2007	1.20 (0.76-1.89)	0.433
2016-2023 vs 2000-2007	1.03 (0.62-1.70)	0.923
**Attained age (x 10 years more)**	1.12 (0.95 -1.32)	0.169
**HIV risk group**		
PWID vs HE	2.79 (1.78-4.36)	<.0001
MSM vs HE	1.50 (0.92-2.43)	0.103
Other/unknown vs HE	1.59 (0.66-3.84)	0.305
**Prior AIDS diagnosis, yes vs no**	1.76 (1.20 -2.60)	0.004

Supplementary Table 6. Multivariable Poisson regression models of risk of development virus-related cancers.

List of abbreviations: aRR, adjusted relative risk; CI, confidence interval; PWID, people who inject drugs; HE, heterosexual; MSM, man who have sex with man.

	aIRR (95% CI)	p
**Period**		
2008-2015 vs 2000-2007	0.85 (0.40-1.84)	0.686
2016-2023 vs 2000-2007	1.45 (0.68-3.05)	0.334
**Attained age (x 10 years more)**	1.50 (1.20-1.88)	0.0005
**HIV risk group**		
PWID vs HE	1.08 (0.56-2.10)	0.812
MSM vs HE	0.59 (0.28-1.24)	0.164
Other/unknown vs HE	0.77 (0.19-3.23)	0.724
**Prior AIDS diagnosis, yes vs no**	1.33 (0.72-2.45)	0.364

Supplementary Table 7. Multivariable Poisson regression models of risk of development lung cancers.

List of abbreviations: aRR, adjusted relative risk; CI, confidence interval; PWID, people who inject drugs; HE, heterosexual; MSM, man who have sex with man.

	aIRR (95% CI)	p
**Period**		
2008-2015 vs 2000-2007	1.87 (0.86-4.09)	0.115
2016-2023 vs 2000-2007	0.75 (0.26-2.18)	0.595
**Attained age (x 10 years more)**	1.50 (1.00-2.25)	0.052
**HIV risk group**		
PWID vs HE	11.16 (4.28-29.08)	<.0001
MSM vs HE	1.44 (0.42-5.01)	0.563
Other/unknown vs HE	.	.
**Prior AIDS diagnosis, yes vs no**	0.63 (0.28-1.43)	0.269

Supplementary Table 8. Multivariable Poisson regression models of risk of development hepatocarcinoma.

List of abbreviations: aRR, adjusted relative risk; CI, confidence interval; PWID, people who inject drugs; HE, heterosexual; MSM, man who have sex with man.

	aIRR (95% CI)	p
**Period**		
2008-2015 vs 2000-2007	0.83 (0.34-2.03)	0.690
2016-2023 vs 2000-2007	1.50 (0.69-3.25)	0.304
**Attained age (x 10 years more)**	1.07 (0.84-1.37)	0.590
**HIV risk group**		
PWID vs HE	1.13 (0.46-2.75)	0.791
MSM vs HE	2.20 (1.06-4.59)	0.035
Other/unknown vs HE	0.76 (0.10-5.91)	0.796
**Prior AIDS diagnosis, yes vs no**	4.07 (2.11-7.86)	<.0001

Supplementary Table 9. Multivariable Poisson regression models of risk of development anal cancer.

List of abbreviations: aRR, adjusted relative risk; CI, confidence interval; PWID, people who inject drugs; HE, heterosexual; MSM, man who have sex with man.

Cancer site	Sex	Primary analysis: overall cohort SIR (95% CI)	Sensitivity analysis: Milan metropolitan residents SIR (95% CI)
**Lung cancer**	Female	3.21 (1.94-5.32)	3.19 (1.77-5.77)
	Male	1.12 (0.81-1.55)	1.26 (0.89-1.78)
**Anal cancer**	Female	18.55 (8.33-41.30)	25.27 (11.35-56.25)
	Male	59.16 (42.06-83.22)	55.36 (37.11-82.60)
**Hepatocellular carcinoma**	Female	6.95 (2.89-16.69)	7.25 (2.72-19.32)
	Male	3.72 (2.67-5.18)	3.79 (2.61-5.48)

Supplementary Table 10. Standardized incidence ratios (SIRs) for selected cancers in the overall cohort and in the sensitivity analysis restricted to participants residing in the Metropolitan City of Milan. Estimates are shown with 95% confidence intervals.

List of abbreviations: CI, confidence interval; SIR, standardized incidence ratio.
